# Qualitative perspectives of primary care providers who treat Medicaid managed care patients

**DOI:** 10.1186/s12913-018-3516-9

**Published:** 2018-09-21

**Authors:** Sarah H. Gordon, Emily A. Gadbois, Renee R. Shield, Patrick M. Vivier, Chima D. Ndumele, Amal N. Trivedi

**Affiliations:** 1 Department of Health Services, Policy, and Practice, Brown University, 121 South Main Street, Providence, RI 02912 USA; 2 Hassenfeld Child Health Innovation Institute, Brown University, Providence, RI USA; 3 Department of Health Policy and Management, Yale University School of Public Health, New Haven, CT USA; 4 Providence VA Medical Center, Providence, RI USA

**Keywords:** Qualitative, Primary care, Medicaid, Managed care, Vulnerable populations

## Abstract

**Background:**

Declining job satisfaction and concurrent reductions in Medicaid participation among primary care providers have been documented, but there is limited qualitative work detailing their first-hand experiences treating Medicaid patients. The objective of this study is to describe the experiences of some primary care providers who treat Medicaid patients using in-depth qualitative analysis.

**Methods:**

We conducted qualitative interviews with 15 primary care providers treating Medicaid patients in a Northeastern state. Participant recruitment efforts focused on including different types of primary care providers practicing in diverse settings. Qualitative interviews were conducted using a semi-structured interview protocol. We developed a coding scheme to analyze interview transcripts and identify themes.

**Results:**

Providers expressed challenges effectively meeting their patients’ needs under current policy. They described low Medicaid reimbursement and underinvestment in care coordination programs to adequately address the social determinants of health. Providers shared other concerns including poor access to behavioral health services, discontinuous Medicaid coverage due to enrollment and renewal policies, and limited reimbursement for alternative pain treatment. Providers offered their own suggestions for the allocation of financial investments, Medicaid policy, and primary care practice.

**Conclusions:**

Underinvestment in primary care in Medicaid may detract from providers’ professional satisfaction and hinder care coordination for Medicaid patients with complex healthcare needs. Policy solutions that improve the experience of primary care providers serving Medicaid patients are urgently needed to ensure sustainability of the workforce and improve care delivery.

## Background

Primary care physicians play an integral role in the healthcare system by providing first-contact, comprehensive, ongoing, and integrated care. Primary care services enhance timely access to care, improve health outcomes, and lower healthcare costs, and are particularly critical to the networks of care that support low-income Medicaid populations and reduce health disparities [1–8]. Despite the importance of primary care providers to the Medicaid program, provider participation rates have historically been low [9]. In 2011, nearly one-third of physicians were unwilling to accept new Medicaid patients. Providers decline Medicaid insurance for a variety of reasons, from concerns regarding taking on the risk of caring for complex patients to the administrative burden of navigating a state-run insurance program [10]. In addition, low reimbursement rates and delays in payment have repeatedly been shown to discourage providers from accepting Medicaid [11–14].

There is limited qualitative work focusing on the first-hand experiences of physicians who deliver care to the Medicaid managed care population [15–17]. Physician perspectives are rarely considered in administrative strategies, despite the reliance on primary care in many cost-containment and quality improvement programs at the state and federal levels. Previous qualitative studies of physicians’ experiences treating Medicaid managed care patients were published over 15 years ago after the initial growth of managed care [18]. Over the past several decades, Medicaid managed care has grown rapidly, and on average, 77% of current state Medicaid beneficiaries are enrolled in managed care [19, 20].

In this study, we conducted in-depth interviews with primary care providers delivering care in a state with high Medicaid managed care penetration and comparatively low Medicaid reimbursement relative to other states. The objective was to illuminate the experiences of primary care providers treating Medicaid managed care patients.

## Methods

### Study design/sample

Semi-structured qualitative interviews were conducted between February and October of 2016 with 15 physicians who provide primary care to Medicaid managed care beneficiaries in a Northeastern state. These interviews were part of a broader mixed-methods evaluation of a natural experiment in which the state randomly assigned Medicaid beneficiaries to the remaining managed care plans following the exit of another plan. Our interview protocol asked providers questions regarding quality improvement in Medicaid managed care, but a large portion of physician responses focused on their own experiences. The interview protocol is available as an Appendix accompanying this manuscript.

Participants included eleven pediatricians (of which two were residents), one pediatrician/internal medicine physician, one family physician, and two obstetricians. Practice settings included small private practices, community health centers, and large hospital-based clinics. Two providers reported practicing in patient-centered medical home settings. The proportion of providers’ patient panels enrolled in Medicaid varied from 5 to 90%.

Providers were recruited via email, physician email listservs, and state provider organizations. We complemented these methods with snowball sampling to invite additional providers to participate. Recruitment of providers used purposive sampling to prioritize equal numbers of different primary care specialties. Eligibility criteria were restricted to primary care providers who treated Medicaid patients. All participants were compensated with a $50 gift card. This study was reviewed and exempted by the Brown University Institutional Review Board. We utilized the Consolidated Criteria for Reporting Qualitative Studies (COREQ) checklist to guide our study design, data collection, and analysis.

### Data collection

Three female researchers with extensive backgrounds in qualitative research and doctorates in relevant fields conducted the qualitative interviews. The methodological approach was a content analysis with a grounded theory orientation that enabled us to identify themes that were not asked about or expected. Provider interviews were semi-structured and asked about providers’ practices, important quality measures, and differences between managed care plans’ approaches to quality management for children and women of child-bearing age. The protocol was piloted with three providers and refined to enhance clarity. Telephone interviews lasted between 30 and 60 min. With participant consent, all interviews were audio recorded and subsequently transcribed for analysis.

### Analysis

A coding scheme was developed by the 5-member analysis team after reviewing the first two transcripts, and was refined through subsequent group discussion. Transcripts were then assigned to teams of two or three that rotated throughout the coding process. Each researcher in the subgroup coded each transcript independently and then met within the subgroups to reconcile their decisions. At regular intervals, the full analysis team met to discuss potential themes that were emerging and to update the coding scheme if necessary. An audit trail recorded coding definitions and decisions and noted discussions about emerging themes [21]. Coded transcript data were organized using the qualitative software package NVivo, version 11.

## Results

Descriptive statistics of the provider respondents are provided in Table 1. During analysis of the physician interviews, distinct themes arose that were independent from the original goal of the interviews (to assess quality management strategies employed by managed care organizations) and were shared across participants. In general, providers described challenging experiences meeting the needs of their Medicaid patients and described a strained relationship with Medicaid managed care. Providers discussed barriers to addressing the complex healthcare needs of their Medicaid patient populations and expressed concerns regarding the viability of treating Medicaid patients under current reimbursement rates. Physicians also commented on a range of Medicaid policies, including enrollment and renewal processes, prescription drug coverage, and access to behavioral health services that impact the health and well-being of Medicaid patients. Finally, physicians offered suggestions for improvements and highlighted policies that could be reformed to improve the experiences of both providers and patients in the Medicaid program.

**Table 1:** Characteristics of Primary Care Provider Participants, *N* = 15

Variable	*N* (%)
**Provider Type**	
Pediatrician	11 (73.3)
Family Physician	1 (6.7)
Internal Medicine/Pediatrics	1 (6.7)
Obstetrics/Gynecology	2 (13.3)
**Practice Setting**	
Small private practice	6 (42.8)
Large, hospital-based practice	8 (57.1)
**Proportion of Patient Population Receiving Medicaid Benefits**	
0–25	5 (33.3)
25–50%	4 (26.7)
50–75%	1 (6.7)
75–100%	5 (33.3)

Note: Information on practice setting was not obtained for one participant

### Theme 1. Addressing the social determinants of health and complex healthcare needs of Medicaid patients is challenging

Providers described challenges meeting patient needs that are inadequately addressed by traditional health care services. Specific concerns raised in the interviews included transportation, safety in the home, drug use, nutrition, housing, and the lack of economic opportunity. In the words of one provider:

“[M]ost important for pregnant women … [and] for kids … [are] the social determinants of health -- … healthy food, clothing, education, money for the basics, those things which are not specific to health care but are really specific to health.”

*-Pediatrician #1*

Physicians commented on difficulties their patients face in obtaining time off from work and childcare required to attend office visits. Providers also referenced the challenges of maintaining care plans with patients with language barriers and/or low levels of reading comprehension. One obstetrician stated:

“One of the greatest predictors of outcome is just that, health literacy. You can make all the diagnoses you want. You can do all the treatment you want. But if the patient does not understand what’s happening with them, and doesn’t understand the treatment that they are supposed to go through then you have a greater number of complications and a greater number of people not following through or following up.”

*- Obstetrician #1*

Pediatrician participants consistently commented on the importance of school attendance, child reading level, and expressed challenges supporting and monitoring these foundations of children’s health that occur beyond the clinic:

“Again I know that’s not a specific purview of a health insurance company but if there’s a way, that’s a huge barometer of family health and patient health if they’re able to get out the door to school … how can we put systems in place in a home so that family is able to get the children out to school every single day.”

-*Pediatrician #2*

### Theme 2. Low reimbursement rates and inadequate investment in quality management initiatives limit care coordination and the number of low-income patients providers treat

Though we did not specifically ask about financial reimbursement, participants brought up the challenges of serving Medicaid patients under current reimbursement rates and the inadequacy of investment in care coordination. One pediatrician in a small practice said that they were no longer accepting any new Medicaid patients because the reimbursement for seeing Medicaid patients did not cover office overhead. While praising the expansion of Medicaid eligibility for low-income residents, another physician expressed concerns regarding provider shortages to meet the needs of the newly-enrolled. This provider also described the impact of reimbursement changes on personal finances:

“In the last eighteen months … they lowered the reimbursement… actually below those that were established before Obama had increased them to Medicare reimbursement, so we’re suffering a double hit… The fees only cover the cost of running the office so I’m running in step. It’s a really furiously bad situation…The more poor people you see, the less money you make.”

*-Pediatrician #3*

Physicians who discussed incentives for care coordination expressed approval of even modest funds to coordinate care for complex patients. However, providers who discussed these payments unanimously commented on their inadequacy to produce meaningful results. One provider further stated that the documentation required to participate in quality management initiatives outweighed the financial incentive:

“There are some incentive programs that we participate in and we see a little bit of extra money from, to be candid there’s so much book work, it’s sort of a wash because the staff have to do so much, paperwork to document all of this stuff and fill in all this stuff, I’m not sure that we actually make money off of it… I don’t see it as being a profit center.”

-*Pediatrician #3*

### Theme 3. Specific Medicaid policies are perceived as detrimental to patient outcomes and quality of care

Physician participants perceived that Medicaid policies adversely affected their ability to deliver effective patient care, citing behavioral health, continuity of Medicaid enrollment, and access to medications for chronic pain management as important concerns. Example quotations are provided in Table 2. Providers lamented the lack of integration for children’s behavioral health services, describing how access to mental health services is limited by complex benefit design, poor mental health provider participation, and underfunding. Three providers described specific difficulties their patients had making an appointment with a mental health professional. One of whom described inadequate access to outpatient behavioral health services:

“They have very poor access…[to] mental health services. The mental health services they *do* have, on paper don’t seem to solve the problems…There’s a *handful* that take insurance…but it’s with outpatient behavioral mental health services …it’s the wild west and sometimes not even that for families [with] Medicaid.”

-*Pediatrician #4*

**Table 2:** Theme 3 Quotations on the Impact of Medicaid Program Structure on Patient Outcomes

*Behavioral Health*	“Children’s behavioral health is spread over five different state agencies…and although there are some efforts toward behavioral health integration, even now, within Medicaid managed care, mental health is a carve-out. But that’s ridiculous. Children’s behavioral health is tightly tied to their whole health, and to have a system where…the head [is] in one area and the body in another area, and ability to co-manage, co-care for, co-locate so they sort of integrate care is, is hamstrung by, regulatory issues, and Medicaid is just, it’s ridiculous.” -*Pediatrician #5*
*Continuity of Medicaid Enrollment*	“But I think one of the other *really* big issues that doesn’t get a lot of play is the fact that for a lot of my patients once their pregnancy is completed, they lose insurance…They either have to reenroll or they’re not eligible for reenrollment. And that happens about 6 weeks, 5 weeks now, after their birth. So that’s a real challenge…making sure that patient comes in for a postpartum visit is key and providing sustainable contraception is key. I mean, that’s preventative care at its best…And, and to not have…the benefits is really [a] problem.” -*Obstetrician #2*
*Pain Management Medications*	“I cannot get chiropractic care, I cannot get acupuncture, I cannot get anything that isn’t pills. Throw into that…you won’t let me prescribe anything other than narcotics for the most part because their cheap, and then you’re telling me I’m prescribing too many narcotics.” -*Family Physician #1*

Providers also described how Medicaid eligibility and enrollment policies affect continuity of care, delivery of preventive care, and physician reimbursement. One obstetrician discussed how Medicaid coverage loss for pregnant women 60 days after delivery impedes basic postnatal care. This obstetrician described how his/her practice has altered the postnatal care regimen to fit within the post-partum Medicaid coverage period. Another provider discussed how if patients do not re-enroll in Medicaid coverage at regular intervals, the family is automatically dropped from coverage, resulting in foregone reimbursement. Finally, a family physician explained how despite administrative pressure to reduce opiate prescriptions to treat chronic pain, Medicaid does not cover alternative pain treatments.

### Theme 4. Providers offer suggestions for how to improve care delivery and reform Medicaid policy

Providers offered specific suggestions to address the social determinants of health. Providers recommended home-based risk assessments and interventions delivered by nursing staff. One provider suggested treating both mothers and children at the same visit and treating the family as a unit:

“I think it would be great to have Medicaid initiatives that focus on a family centered approach, or for example, if obesity is an issue, if the child is facing or is at risk for childhood obesity and childhood diabetes, most likely other people in the family [are] as well.”

*-Family Medicine Resident #1*

One pediatrician proposed that school attendance be included as a standard measure of children’s health, and a different pediatrician pushed for screening for behavioral changes and stressors in the home because “that’s where we can really make change in children’s lives and families’ lives.” Another provider explained how carefully examining risk factors could help predict resource needs:

“Looking at...families that have more than two addresses in 24 months, or families who have three children under 5 or 4 years of age—we have the ability [and] if we could look at that data [we could] do a much better job reaching out to families in a preventive way.”

*-Pediatrician #5*

Despite providers’ complaints regarding financial reimbursement, physicians described a moral commitment to serve Medicaid patients and offered suggestions for improvement. Providers recommended per-member per-month care coordination investment, funding for non-medical staff to carry out care coordination responsibilities, and consolidating investments in children’s health across state agencies to improve efficiency of existing efforts.

Regarding access to behavioral health services, one provider suggested that an on-site mental health practitioner could help patients who may be discouraged by the barriers of navigating a complex system:

“We've done so much better with places…when they are willing to provide support for someone who could be on site to meet the person and actually start triaging them and introduce them to someone directly that [patients] are less intimidated by. The process works much better.”

-*Pediatrician/Internist #6*

Providers highlighted specific eligibility and renewal policies that they deemed as harmful to continuity of care. Providers encouraged extending the eligibility period for pregnant women to align with the timeline of recommended post-partum care and the adoption of simplified enrollment procedures such as automatic renewals or continuous eligibility policies to reduce lapses in reimbursement for providers and insurance coverage for patients. Finally, one provider encouraged expanding reimbursement for non-opioid pain treatments.

## Discussion

In this exploration of Medicaid providers’ perceptions of care delivery, providers noted important challenges in treating the complex needs of Medicaid patients. They expressed frustration with current Medicaid reimbursement rates and inadequate investment in programs to coordinate care for Medicaid patients and address social determinants of health. Providers discussed other concerns pertaining to state Medicaid policies, including the lack of behavioral health integration and poor access to behavioral health services, Medicaid enrollment and renewal policies that erode continuity of care, and the lack of alternative treatments for chronic pain approved by Medicaid. However, providers also offered insightful strategies to improve care for Medicaid patients.

Providers’ descriptions of the challenges in caring for the complex needs of Medicaid patients and onerous administrative requirements align with prior work, suggesting there have not been significant improvements over the past several decades [22]. Projections show that insurance expansions under the ACA will require 52,000 additional primary care physicians by 2025 [23]. The experiences of provider participants regarding Medicaid reimbursement reflect state-level policy: these providers practice in the state with the lowest ratio of Medicaid to Medicare payments in the country (0.38 for all services, 0.33 for primary care, and 0.41 for obstetric care). In 2012, Medicaid paid only 66% of Medicare rates nationally [24]. In response to these trends, the ACA temporarily increased Medicaid reimbursement rates to Medicare levels to encourage provider participation to meet the demand of new Medicaid enrollees. After the payment increase, the availability of primary care appointments increased 7.7%, with the largest increases in states that had lower initial reimbursement rates [25]. However, only 15 states have opted to continue funding primary care reimbursement at ACA rates, and as of July 2016, nation-wide average Medicaid reimbursement rates had declined to 72% of Medicare rates [26].

While citing significant challenges, providers offered important policy recommendations that could enhance access, efficiency, and quality of care through programmatic and policy changes that span from the office setting to the plan level. Suggestions included combining parent-child visits, extending the Medicaid eligibility period for pregnant women to include the standard postpartum care timeline, providing on-site mental health consultations to facilitate connection to behavioral health services, and in-home risk assessments of the social determinants of health administered by non-physician staff. These suggestions underscore providers’ abilities to come up with creative solutions and demonstrate how incorporating provider feedback and facilitating engagement between providers, health plans, and Medicaid administrators could potentially improve outcomes for Medicaid beneficiaries.

A limitation of this qualitative study is that we interviewed a sample of 15 physicians who practice in one state. However, this study was not intended to generalize to all primary care providers who serve Medicaid patients. Providers who experience the most extreme working conditions may also have been the most eager to share their thoughts, resulting in a potentially biased representation of primary care practice. Nonetheless, because these issues were raised by a diverse group of providers without prompting, we believe they reflect important insights into the experiences of the primary care workforce.

## Conclusions

In this qualitative study of primary care physicians’ perceptions of providing care to Medicaid patients, participants described substantial challenges and suggestions for improvement in serving a patient population with complex and unmet social and healthcare needs. Low reimbursement, inadequate financial resources, limited access to behavioral health care, and poorly designed Medicaid coverage policies reduced providers’ professional satisfaction and the quality of care. Policy solutions that to improve the experience of primary care providers serving Medicaid patients are urgently needed to ensure sustainability of the workforce and improve care delivery.

## Appendix

### Appendix 1. Health Care Provider Interview Protocol

Thank you for speaking with me. The goal of our research is to identify successful initiatives and strategies for improving the quality of care and health outcomes of children and women of child-bearing age enrolled in Medicaid. During our research, we will be speaking to health plan representatives, state Medicaid officials and healthcare providers to better understand the system of care in place for these populations. Today, we are interested in your experiences working with Medicaid managed care plans and are especially interested in knowing how they are working to improve care quality and outcomes for these populations.

1. Please begin by describing your practice. What proportion of your patients are enrolled in Medicaid?

2) What care quality and outcome measures do you think are most important to the health of children in the [state] Medicaid program?

3) What care quality and outcome measures do you think are most important to the health of pregnant women in the [state] Medicaid program? What about women of childbearing age?

4) What are your perceptions about the strategies that Medicaid managed care plans employ to promote quality and improve outcomes for children and women of child-bearing age?

- Please tell us how well you think these are working.
- What hasn’t worked?
- Tell us about other things you think could be tried to improve quality and outcomes.
- Do they differ for Plan A versus Plan B?
- (if a quality improvement strategy by Plan A or Plan B is mentioned) then ask

“What impact did that strategy have on the patients of that plan? Did the quality improvement strategy impact the patients of other plans? If so, How?”

5) Do managed care plans provide you or your patients with any promotional or informational materials?

- If so, have any of these been related to preventative healthcare guidelines for children or expectant mothers?
- Do they differ for Plan A versus Plan B?
- Have these materials worked to improve care quality?

6) Please describe any incentive programs Medicaid managed care plans have in place to promote adherence to preventative healthcare guidelines for children or women?

- Do they differ for Plan A versus Plan B?
- Have these incentives worked?

7) Have managed care organizations communicated any specific care quality or health outcome goals for these populations to you?

- If so, how this was achieved?
- Who was involved?
- Do they differ for Plan A versus Plan B?

8) Do managed care plans provide you with any data about care quality or the health outcomes of enrollees?

- Are these data are provider-specific? Condition-specific?
- Does this differ for Plan A versus Plan B?
- Does the provision of this data work to improve care quality or health outcomes?

9) Do you have any suggestions about how Medicaid managed care plans could improve the care quality and health outcomes experienced by children or women of child-bearing age?
10) That was my last question, but is there anything you would like to add about this topic that I’ve not asked about?
11) Can you suggest anyone else that we should speak to?
12) Address to send gift card:

### Appendix 2. Coding Scheme

1. General

A. Description of provider’s practice
B. Plan relationships with FQHCs
C. Big picture ideas/problems
D. Other

2. Medicaid/Managed Care Relationships

A. Description of market
B. How Medicaid identifies, contracts with, and oversees plans
C. Why insurers choose to leave market
D. Assignment of enrollees
E. Other

3. Measures

A. Measures considered important for patients in [state] Medicaid program
B. Why measures were selected
C. Data that the Medicaid Office generates regarding these measures
D. Perceived strengths and weaknesses of measures
E. Other

4. Strategies Managed Care Plans Employ to Improve Quality

A. Descriptions of strategies targeting providers
B. Descriptions of strategies targeting patients
C. Strengths/challenges/effects
D. Ideas for improvement
E. Plan A vs. Plan B
F. Other

5. Data

A. How managed care organizations collect data
B. How data is used by MCOs
C. Data attribution
D. Strengths/weaknesses
E. Other

6. Good Quote
